# Radio Frequency Signal Recognition of Unmanned Aerial Vehicle Based on Complex-Valued Convolutional Neural Network

**DOI:** 10.3390/s26020620

**Published:** 2026-01-16

**Authors:** Yibo Xin, Junsheng Mu, Xiaojun Jing, Wei Liu

**Affiliations:** 1 School of Information and Communication Engineering, Beijing University of Posts and Telecommunications, Beijing 100876, China; hszxyb21@bupt.edu.cn (Y.X.); jxiaojun@bupt.edu.cn (X.J.); 2 School of Intelligent Engineering and Automation, Beijing University of Posts and Telecommunications, Beijing 100876, China; twhlw@163.com

**Keywords:** unmanned aerial vehicle, radio frequency, complex-valued convolutional neural network, Sobel edge detection, ablation experiments

## Abstract

The rapid development of unmanned aerial vehicle (UAV) technology necessitates reliable recognition methods. Radio frequency (RF)-based recognition is promising, but conventional real-valued CNNs (RV-CNNs) typically discard phase information from RF spectrograms, leading to degraded performance under low-signal-to-noise ratio (SNR) conditions. To address this, this paper proposes a complex-valued CNN (CV-CNN) that operates on a constructed complex representation, where the real part is the logarithmic power spectral density (PSD) and the imaginary part is derived from Sobel edge detection. This enables genuine complex convolutions that fuse magnitude and structural cues, enhancing noise resilience. As complex-valued networks are known to be sensitive to architectural choices, we conduct comprehensive ablation experiments to investigate the impact of key hyperparameters on model performance, revealing critical stability constraints (e.g., performance collapse beyond 4–5 network depth). Evaluated on the 25-class DroneRFa dataset, the proposed model achieves 100.00% accuracy under noise-free conditions. Crucially, it demonstrates significantly superior robustness in low-SNR regimes: at −20 dB SNR, it attains 15.58% accuracy, over seven times higher than a dual-channel RV-CNN (2.20%) with identical inputs; at −15 dB, it reaches 45.86% versus 14.03%. These results demonstrate that the CV-CNN exhibits potentially superior robustness and interference resistance in comparison to its real-valued counterpart, maintaining high recognition accuracy even under low-SNR conditions.

## 1. Introduction

Reliable detection and recognition of UAVs are critical for ensuring safety and security in low-altitude airspace [1]. Existing detection technologies face significant limitations: acoustic sensors suffer from short range and ambient noise interference [2], visual sensors are affected by weather and lighting conditions [3], and radar systems, while robust, are costly and prone to clutter from obstacles [4]. Recent work has also explored novel sensing modalities such as electrostatic charge accumulation on the UAV surface during high-speed flight [5] and vibration signatures induced by propeller rotation, which can be measured via radar for fine-grained identification and tamper detection [6]. Furthermore, system-level solutions leveraging secure drone-to-drone communication and blockchain-based traffic management have been proposed to address safety and privacy concerns from a different angle [7]. RF-based detection has thus emerged as a promising alternative due to its long range, all-weather capability, and ability to identify RF-emitting UAVs even when visually obscured [8].

The efficacy of deep learning in UAV RF signal recognition has been widely demonstrated [9,10,11,12], with CNNs becoming the mainstream approach [13,14,15,16,17]. A study by O’Shea et al. [18] demonstrated the effectiveness of CNNs for radio modulation recognition, laying the groundwork for applying deep learning to RF signal analysis. However, most RV-CNNs process only the magnitude component of the RF spectrogram, discarding phase information that encodes critical hardware-specific features such as oscillator nonlinearity and clock offsets [19]. This omission compromises signal integrity and degrades recognition performance, particularly under low-SNR conditions where phase cues are essential for discrimination [20].

In view of the statistical correlation that characteristically exists between the real and imaginary parts of complex numbers, the utilization of complex-valued modeling approaches is more substantiated. Complex-valued systems impose stronger mathematical constraints than real-valued systems, thereby facilitating the extraction of more intrinsic feature representations [21]. A comprehensive survey on complex-valued neural networks can be found in [22], which details their theoretical advantages and diverse applications. Despite the existence of studies that propose the decomposition of complex values into two real components for the purpose of separate processing, the supposition that a CV-CNN is equivalent to a two-dimensional RV-CNN is erroneous. Research [23] demonstrated that the computational constraints introduced by complex multiplication limit the freedom of CV-CNNs in synaptic weighting, resulting in a fundamental difference in representational capability compared to RV-CNNs.

The employment of CV-CNN ensures the preservation of the algebraic structure inherent within complex data, thus indicating its potential for facilitating the acquisition of more sophisticated feature representations. Extensive research indicates that CV-CNN [24] outperforms traditional RV-CNN [25,26,27] across multiple tasks. However, its adoption remains limited, partly due to the maturity of complex-valued convolutional techniques lagging behind real-valued methods. In recent years, the feasibility of constructing and training CV-CNN has been significantly enhanced by mainstream frameworks such as PyTorch, which have begun to support complex-valued convolutional operations [28]. On this basis, this study proposes a CV-CNN model for recognizing UAV RF signals.

The primary contributions of this work are structured into three core aspects:

(1) Empirical validation of complex-valued deep learning for UAV RF signal recognition: We demonstrate that a CV-CNN, operating on a carefully constructed complex representation, achieves significantly superior robustness under low-SNR conditions compared to a real-valued counterpart with identical architecture and input. This provides compelling evidence for the practical advantage of complex-domain modeling in RF-based UAV recognition, a domain where phase-like structural cues are critical for noise resilience.
(2) A hybrid complex feature engineering strategy: Recognizing that the raw RF phase is lost during PSD computation, we propose a pragmatic surrogate for the imaginary component by applying Sobel edge detection to the logarithmic PSD. This design fuses magnitude and structural information into a complex-valued input, enabling genuine complex convolutions that prove highly effective for classification in noisy environments, as validated by our experiments.
(3) Comprehensive ablation and comparative analysis: We conduct a systematic investigation into the sensitivity of CV-CNN performance to key hyperparameters (network depth, kernel size, and dropout strategy), revealing critical stability constraints (e.g., performance collapse beyond 4–5 layers). Furthermore, we perform a fair, controlled comparison against a dual-channel real-valued CNN (dual-RV-CNN) using the exact same input features, conclusively attributing the low-SNR advantage to the complex arithmetic itself rather than the input representation.

Compared to existing studies, our work presents distinct advancements. First, unlike conventional RV-CNN approaches [13,14,15] that operate solely on magnitude spectrograms and discard phase-related information, our CV-CNN explicitly models the coupling between magnitude and structural cues via complex arithmetic, yielding significantly improved low-SNR robustness. Second, while prior complex-valued models in RF classification (e.g., [27]) typically require access to the full complex time–frequency representation (e.g., raw I/Q data or complex STFT) to exploit phase information, our method is uniquely designed for practical scenarios where only the power spectral density (PSD) is available. By ingeniously substituting the missing phase with Sobel-derived structural features, we retain the benefits of complex-domain processing without relying on raw signal access. Third, in contrast to generic deep architectures like ResNet18 or EfficientNet-B0 that treat RF spectrograms as ordinary images [8], our CV-CNN is purpose-built for the algebraic structure of RF signals, as evidenced by its superior performance under noise.

The remainder of this paper is organized as follows: Section 2 introduces the system model. Section 3 provides a detailed description of the proposed CV-CNN model. In Section 4, the experimental methodology, configuration, and ablation results on the DroneRFa dataset are presented. Section 5 offers a dedicated discussion that contextualizes the findings, compares them with the relevant literature, and critically analyzes the limitations of the proposed approach. Finally, we conclude our work in Section 6.

## 2. System Model

**Figure 1:**
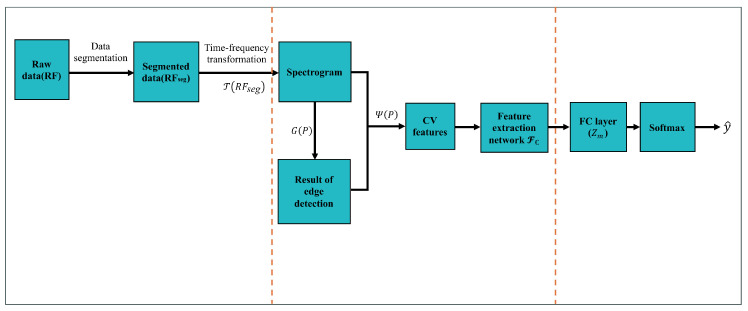
The proposed system model.

The proposed system model, as shown in Figure 1, is intended to address the classification of RF signals from UAVs, with the objective of achieving this aim through the implementation of a three-stage processing framework. The initial section, Section 2.1, provides an exposition of signal preprocessing and time–frequency analysis. The subsequent section, Section 2.2, introduces complex-valued feature extraction methods. Finally, Section 2.3 details the classification and decision process.

### 2.1. Signal Preprocessing and Time–Frequency Analysis

The RF signal data used in this study are drawn from the publicly available DroneRFa dataset [29], which contains raw RF recordings from several distinct categories of commercially available UAVs. The dataset encompasses a diverse range of UAV models from various manufacturers, including but not limited to DJI (e.g., Mini 2, Mavic Pro, Phantom 4 Pro, Inspire 2), and mid-sized platforms such as the MATRICE series. The data are collected using an NI (National Instruments, Austin, TX, USA) USRP-2955 RF transceiver and VERT2450 omnidirectional gain antennas, and a host computer. The USRP-2955, which features four RF channels and supports a sampling rate of up to 100 MS/s, is used to capture the complex I/Q data. The VERT2450 antennas, with a gain of 3 dBi, are tuned to common UAV control and video transmission bands (2.4–2.48 GHz and 4.9–5.9 GHz). The host computer communicates with the USRP via a PCIe interface, ensuring high-throughput data transfer.

The input data comprises dual-channel raw RF data stored in .mat files. The present dataset comprises the in-phase components ($\mathrm{RF}0_{I}$, $\mathrm{RF}1_{I}$) and the quadrature components ($\mathrm{RF}0_{Q}$, $\mathrm{RF}1_{Q}$), with the $‘\mathrm{RF}0^{\prime }$ channel data originating from the first channel of the RF receiver and the ‘RF1’ channel data from the second channel. The letter ‘I’ is used to denote the in-phase component value of the baseband signal, while ‘Q’ is used to denote the quadrature component value. The I/Q components are then combined to form the analyzed signal, which can be written as

(1) $$\begin{array}{cc} RF0 & =RF0_{I}\;+\;j\;\cdot \;RF0_{Q} \end{array}$$

(2) $$\begin{array}{cc} RF1 & =RF1_{I}\;+\;j\;\cdot \;RF1_{Q} \end{array}$$

Then, the continuous IQ data stream undergoes fixed-length segmentation, with each segment comprising 5 million sample points. This segment length was chosen based on two practical considerations. First, the raw RF files in the DroneRFa dataset typically contain over 150 million samples (corresponding to more than 1.5 s of signal), which is too long for direct neural network processing and contains multiple flight states. Second, prior studies have shown that critical RF fingerprinting features of UAVs, such as frequency hopping, transient bursts, and modulation transitions, often occur within time scales shorter than 5 ms [14,20]. A 50 ms segment (5 million samples at 100 MHz) thus provides a sufficient temporal window to capture multiple such transient events while remaining computationally tractable. The 100 MHz sampling rate, fixed during the original data acquisition of the DroneRFa dataset, ensures sufficient bandwidth coverage for the UAV RF bands of interest (2.4–5.9 GHz). Non-overlapping segmentation is adopted to prevent data leakage between training and test samples.

Time–frequency analysis is implemented using the Short-Time Fourier Transform (STFT). For each channel’s data, the STFT results are computed separately using the following equation:

(3) $$\begin{array}{c} S_{0}\left(t,\;f\right)=\mathrm{STFT}\left(RF0_{seg}\right) \end{array}$$

(4) $$\begin{array}{c} S_{1}\left(t,\;f\right)=\mathrm{STFT}\left(RF1_{seg}\right) \end{array}$$

where the STFT results for channels 0 and 1 are denoted by $S_{0}\left(t,\;f\right)$ and $S_{1}\left(t,\;f\right)$. The segmented IQ data for channels 0 and 1 is represented by $\mathrm{RF}0_{\mathrm{seg}}$ and $\mathrm{RF}1_{\mathrm{seg}}$, respectively. Subsequently, the complex STFT results are converted to logarithmic power spectral density $P_{0}$ and $P_{1}$, which can be written as

(5) $$\begin{array}{c} P_{0}=10\log_{10}\left(\right|S_{0}{|}^{2}) \end{array}$$

(6) $$\begin{array}{c} P_{1}=10\log_{10}\left(\right|S_{1}{|}^{2}) \end{array}$$

and the time–frequency transformation operation $T(RF_{\mathrm{seg}})$ can be expressed as

(7) $$\begin{array}{c} T\left(RF_{\mathrm{seg}}\right)=10\log_{10}(|STFT\left(RF_{\mathrm{seg}}\right){|}^{2}) \end{array}$$

This operation computes the logarithmic power spectral density (log-PSD) of the segmented RF signal. Since the STFT itself is a canonical time–frequency representation that captures how the frequency content of a signal evolves over time, the log-PSD inherits this joint time–frequency structure. The logarithmic scaling is a standard practice in RF and audio signal processing, as it enhances the visibility of low-energy components and approximates the human perception of signal power. Thus, T(·) yields a time–frequency representation suitable for visual and machine analysis.

### 2.2. Complex-Valued Feature Construction and Extraction

During the feature construction phase, the raw logarithmic power spectrum $P_{0}/P_{1}$ is initially treated as the real part, while the Sobel edge detection results from the spectral image are computed as the imaginary part. In the specific implementation, the imaginary part feature is generated by

(8) $$\begin{array}{c} G\left(P\right)=\sqrt{{\left(P*S_{x}\right)}^{2}+{\left(P*S_{y}\right)}^{2}} \end{array}$$

where $S_{x}$ and $S_{y}$ denote the horizontal and vertical convolutional kernels of the Sobel operator, respectively, and ∗ represents the convolutional operation. The Sobel operator is a widely used image processing technique for edge detection. It works by computing the image gradient in two orthogonal directions: $S_{x}=\left[\begin{array}{ccc} -1 & 0 & 1\\ -2 & 0 & 2\\ -1 & 0 & 1 \end{array}\right]$ highlights vertical edges by detecting horizontal intensity changes, while $S_{y}=\left[\begin{array}{ccc} -1 & -2 & -1\\ 0 & 0 & 0\\ 1 & 2 & 1 \end{array}\right]$ highlights horizontal edges by detecting vertical intensity changes. The resulting magnitude map G(P) emphasizes regions of high spatial frequency (i.e., edges and texture), which serve as a phase-like structural surrogate in our complex representation. We clarify that the imaginary component G(P) is not the original RF phase but a phase-like surrogate encoding spatial structure via edge gradients. This design reflects our hypothesis that structural features, rather than raw phase, are more discriminative for classification under strong-noise conditions.

The complex-valued feature construction operation, designated as Ψ(P), can be defined as

(9) $$\begin{array}{c} \Psi (P)=P+j\;\cdot \;G(P) \end{array}$$

The feature extraction section comprises *N*, where *N* is an integer no less than 3, cascaded complex convolutional blocks. Each constituent block comprises a complex convolutional layer, a CBN layer, and a CReLU activation layer. The number of channels increases according to the pattern $32\to 64\to \ldots \to 32\;\times \;2^{n-1}$, ultimately resulting in the output of feature maps via adaptive average pooling.

CV-CNN performs feature extraction on G(P) with the complex convolutional operation adhering to the following mathematical definition:

(10) $$\begin{array}{cc} \mathrm{Conv}(X) & =\left(W_{r}+jW_{i}\right)\left(X_{r}+jX_{i}\right)\\ \; & =\left(W_{r}X_{r}-W_{i}X_{i}\right)+j\left(W_{r}X_{i}+W_{i}X_{r}\right) \end{array}$$

where the real and imaginary parts of the convolutional kernel weights are denoted by $W_{r}$ and $W_{i}$, respectively, while $X_{r}$ and $X_{i}$ represent the real and imaginary parts of the input features. CBN of the input features can be defined as

(11) $$\mathrm{CBN}\left(X\right)=\gamma \left(\frac{X_{r}-\mu_{r}}{\sqrt{\sigma_{r}^{2}+\epsilon }}\right)+j\gamma \left(\frac{X_{i}-\mu_{i}}{\sqrt{\sigma_{i}^{2}+\epsilon }}\right)$$

where $\mu_{r}$ and $\mu_{i}$ represent the mean values, $\sigma_{r}^{2}$ and $\sigma_{i}^{2}$ denote the squares of the standard deviations of the real and imaginary parts, and γ is the scaling factor.

The activation function adopts the CReLU form, which involves the application of a ReLU nonlinear transformation to the magnitude of complex signals while preserving phase information. This can be expressed as

(12) $$\begin{array}{c} CReLU\left(X\right)=ReLU\left(|X|\right)\;\cdot \;e^{j∠X} \end{array}$$

where j∠(X) denotes the phase angle of a complex signal.

The complete complex feature extraction network may be expressed as $F_{C}$ over the complex domain C.

(13) $$\begin{array}{c} F_{C}=AP\circ \left(\overset{N}{\underset{k=1}{⨂}}CReLU\circ CBN\circ Conv\right) \end{array}$$

where the symbol ∘ is used to emphasize the directionality of data flow, ⊗ denotes the stacking of convolutional blocks, and AP represents the pooling method. In the case of k<N, AP denotes maximum pooling; in the case of k=N, AP denotes adaptive average pooling.

### 2.3. Classification and Output

The classifier employs a fully connected (FC) layer architecture comprising 1024→512→m neurons, with dropout regularization applied at the 512-dimensional hidden layer at a probability p=0.5. The system’s output layer generates an *m*-dimensional logit vector *Z*, corresponding to binary encodings for *m* distinct UAV types. The classification decision formula may be expressed as

(14) $$\begin{array}{cc} Z_{m} & =FC_{m}\circ {\mathrm{Dropout}}_{0.5}\circ FC_{512}\circ FC_{1024} \end{array}$$

Softmax is selected as the output function, and the final predicted category formula $\hat{y}$ can be defined as

(15) $$\begin{array}{cc} \hat{y} & =\mathrm{argmax}\left(\mathrm{softmax}\left(Z_{m}\right)\right) \end{array}$$

The complete processing chain may be summarized as follows: The raw IQ signal is subjected to STFT analysis, after which complex features are constructed and extracted. These are then fed into a CV-CNN for classification.

The complete signal processing and classification workflow described by this system may be abstracted as a multi-stage composite function transformation. The mathematical essence of this process is to achieve an end-to-end mapping from raw IQ signals to UAV type identification through a cascade of operations across four stages: time–frequency analysis, complex feature construction, feature extraction, and classification decision-making. The system under discussion can be summarized as formula K, which can be written as

(16) $$\begin{array}{cc} K & =Z_{m}\left(F_{C}\circ \Psi \circ T\left(RF_{\mathrm{seg}}\right)\right) \end{array}$$

The system model’s core innovations can be categorized as follows: The preservation of the phase information of RF signals throughout the process is to be achieved via complex-domain operations. Secondly, the optimization design of time–frequency analysis and deep learning is to be synergistic. Thirdly, a hybrid feature engineering strategy is to be employed, combining traditional signal processing (Sobel edge detection) with data-driven methods. This structured representation not only reveals the mathematical essence of the system but also highlights its theoretical advantage in achieving high-precision RF signal recognition under complex electromagnetic environments.

## 3. The Proposed CV-CNN Scheme

CV-CNN is a deep learning architecture that has been specifically designed for the purpose of processing complex-valued data. The present study has demonstrated that the technology under discussion finds extensive applications in fields requiring phase information preservation. Such fields include radar signal processing, medical imaging (e.g. MRI), and wireless communications. The proposed CV-CNN principally comprises a feature extraction module and a classifier module. In comparison to conventional RV-CNN, CV-CNN meticulously adheres to intricate mathematical principles in pivotal operations, thereby ensuring the model’s capacity to efficiently discern the magnitude and phase characteristics of complex data.

### 3.1. Architecture

**Figure 2:**
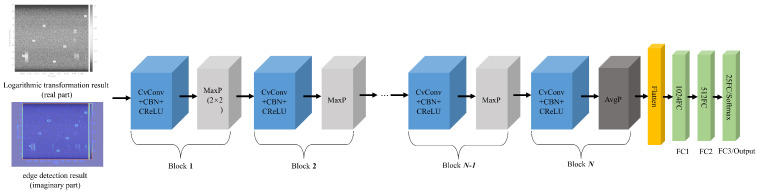
Architecture of the proposed CV-CNN.

The architecture of CV-CNN proposed in this paper represents an enhancement of the model introduced in [30], with its overall structure illustrated in Figure 2. The network under consideration adopts a modular design, primarily consisting of two major modules: a feature extractor and a classifier. The system has been engineered to facilitate efficient complex signal processing through the implementation of meticulously designed complex operation layers.

Within the architecture of the input layer, the network is configured to receive complex tensors of size [batch size, 2, 384, 288]. The first channel is responsible for storing the real-part information of the original spectrogram, while the second channel is responsible for storing the imaginary-part information processed by Sobel edge detection. This dual-channel representation is capable of preserving the complex nature of the signal, thereby establishing the mathematical foundation for subsequent processing.

The feature extractor module consists of N (set to 3–6) stacked complex convolutional blocks, each comprising four key components: The complex convolutional layer employs a four-branch real convolutional function to simulate complex multiplication. The complex arithmetic rule (a+bi)(c+di)=(ac−bd)+(ad+bc)i is implemented rigorously through the parallel branches conv(rr), conv(ri), conv(ir), and conv(ii). This layer is responsible for preserving the spatial dimensions of the feature map through padding operations, whilst only modifying the channel dimension, which may be increased to a maximum of 256 to prevent overfitting and computational explosion. The CBN layer, a key component of the model, jointly normalizes the concatenated real and imaginary parts, thereby preserving the consistency of complex feature distributions. The CReLU activation function is an innovative implementation of a nonlinear transformation in the complex domain. It first calculates the magnitude of the complex number and applies the ReLU operation, and then reconstructs the complex output by integrating the original phase information. The pooling layer employs a hybrid strategy, whereby the first N-1 layers utilize 2 × 2 max pooling to halve the feature map size sequentially, while the final layer switches to 6 × 6 adaptive average pooling to ensure that the network can process inputs of varying resolutions.

We devised two dropout modes (Standard and Reduced) as regularization strategies. The “Standard” mode employs a strategy of progressively increasing dropout rate, raising the rate by 0.1 for each additional convolutional layer (initialized at 0.3). The “Reduced” mode employs the same increment logic for the first three layers (initialized at 0.2); however, it forcibly disables dropout from the fourth layer onwards. Ablation experiments demonstrated that this design effectively balanced the model’s generalization capability and representational power.

The classifier module consists of three FC layers, the function of which is to flatten the four-dimensional feature tensor [batch, C, H, W] into a two-dimensional matrix [batch, C × H × W]. The initial FC layer compresses the high-dimensional features (e.g., 18,432 dimensions) to 1024 dimensions, while the subsequent layer reduces this further to 512 dimensions, ultimately producing 25-dimensional category logits. The implementation of ReLU activation functions and dropout with a 0.5 probability between each FC layer serves to effectively prevent overfitting.

Utilizing a model comprising four convolutional blocks, a kernel size of three, and a dropout pattern of “Reduced” (4L, 3K, Reduced), the model architecture is illustrated in Table 1. The complete forward-propagation process demonstrates the dimensional changes in the data: The input complex signal [batch, 2, 384, 288] undergoes processing through four convolutional blocks, transforming into [batch, 256, 6, 6]. Subsequent to unfolding, this yields a feature vector of [batch, 9216], which is progressively compressed through FC layers to produce an output of [batch, 25]. This architecture design is capable of achieving efficient feature extraction and classification of complex signals while maintaining the mathematical completeness of complex operations.

### 3.2. Feature Extractor

The core components of the feature extractor of the present study comprise complex convolutional layers, CBN layers, CReLU activation functions, and pooling layers, thereby achieving efficient complex signal processing through a modular design.

#### 3.2.1. Complex Convolutional Layer

The core operation of CV-CNN is complex-valued convolution, which is designed to process complex input data. The mathematical foundation of this approach is predicated on the linear separability of complex multiplication, thus exhibiting a fundamental distinction from real-valued convolution, as shown in Table 2.

In the case of an input complex tensor $x\in R^{B\;\times \;2\;\times \;H\;\times \;W}$, where *B* denotes the batch size, 2 represents the real part (channel 0) and the imaginary part (channel 1), and *H* and *W* denote the input height and width, respectively, the tensor can be expressed in this form. It can be demonstrated that, for the complex input z=a+bi and the convolutional kernel $W=W_{r}+W_{i}$, the output $z^{\prime }$ can be expressed as

(17) $$\begin{array}{c} z^{\prime }=\left(a*W_{r}-b*W_{i}\right)+\left(a*W_{i}+b*W_{r}\right)i \end{array}$$

In this context, the symbol ∗ denotes the standard two-dimensional convolutional operation, with $W_{r}$ and $W_{i}$ denoting the convolutional kernel weights for the real and imaginary parts, respectively. In practical implementation, complex-valued convolution is approximated through parallel computation using four real-valued convolutional kernels: ${\mathrm{conv}}_{rr}\left(a\right)$ denotes the convolution of the real part with the real kernel, ${\mathrm{conv}}_{ri}\left(a\right)$ denotes the convolution of the real part with the imaginary kernel, ${\mathrm{conv}}_{ir}\left(b\right)$ denotes the convolution of the imaginary part with the real kernel, and ${\mathrm{conv}}_{ii}\left(b\right)$ denotes the convolution of the imaginary part with the imaginary kernel. The final output is obtained through linear combination:

(18) $$\begin{array}{cc} \mathrm{out}\_\mathrm{real} & ={\mathrm{conv}}_{rr}\left(a\right)-{\mathrm{conv}}_{ii}\left(b\right) \end{array}$$

(19) $$\begin{array}{cc} \mathrm{out}\_\mathrm{imag} & ={\mathrm{conv}}_{ri}\left(a\right)+{\mathrm{conv}}_{ir}\left(b\right) \end{array}$$

This design explicitly models the cross-coupling between the real and imaginary parts, rendering it suitable for time–frequency analysis (e.g., STFT features) and coherent signal processing.

Complex-valued convolution offers several advantages over its real-valued counterpart. Firstly, it preserves phase information, which, in UAV RF signals, carries target motion data, enabling effective modeling of phase variations. Secondly, it exhibits frequency-domain filtering characteristics, with complex kernels functioning as frequency-domain filters that better suit time–frequency analysis tasks (such as STFT feature processing) than real-valued convolution. Thirdly, it offers physical interpretability, as complex operations align with signal processing theory (e.g., analytic signals and Hilbert transform).

#### 3.2.2. CBN Layer

The CBN layer performs independent normalization on the real and imaginary parts to ensure training stability. For complex data z=a+bi, the normalization method is defined as

(20) $$\begin{array}{c} BN(z)=BN(a)+BN(b)i \end{array}$$

where BN denotes a standard BatchNorm2d, which can be expressed as

(21) $$\begin{array}{c} BN\left(X\right)=\gamma \frac{X-\mu }{\sqrt{\sigma^{2}+\epsilon }} \end{array}$$

This specific implementation method involves the calculation of μ and σ separately for the real and imaginary parts. CBN is a method that has been developed to address the issue of internal covariate shifts in complex data. This process prevents optimization difficulties arising from scale differences between real and imaginary components. The primary benefit of this approach is that it stabilizes the distribution of complex features, thereby accelerating the convergence of training. Additionally, it preserves the independence of the real and imaginary components.

#### 3.2.3. CReLU Layer

CReLU is a nonlinear activation function that defines a magnitude activation while preserving the phase in the complex domain. For a complex number z=a+bi, the magnitude |z| and phase ϕ(z) can be written as

(22) $$\begin{array}{c} \left|z\right|=\sqrt{a^{2}+b^{2}+\varepsilon }\;\left(\varepsilon =10^{-6}\right) \end{array}$$

(23) $$\begin{array}{c} \phi (z)=\mathrm{atan}2(b,a) \end{array}$$

The application of a standard ReLU to the magnitude yields $|z^{\prime }|$, which is given by

(24) $$\begin{array}{c} |z^{\prime }\left|=\mathrm{ReLU}(|z|)=\max(0,|z|\right) \end{array}$$

Finally, *z* must be reconstructed in order to derive the CReLU formula:

(25) $$\begin{array}{c} CReLU\left(z\right)=|z^{\prime }\left|\;\cdot \;\cos\phi +i(\right|z^{\prime }\left|\;\cdot \;\sin\phi \right) \end{array}$$

CReLU circumvents the phase distortion issues that arise from conventional real–imaginary separation activation mechanisms by introducing a nonlinear transformation in the vicinity of the origin whilst strictly preserving complex phase information.

#### 3.2.4. Complex Pooling Layer

In CV-CNN, the complex pooling operation is of pivotal importance as a module for the reduction of feature dimensionality, primarily employed to decrease the spatial resolution of feature maps while enhancing translation invariance. In light of the properties inherent to complex data, it is imperative that pooling operations process the real part and the imaginary part separately, thereby ensuring the maintenance of mathematical consistency in complex arithmetic. The specific implementations comprise max pooling and adaptive average pooling.

For the set of complex samples within the sliding window (win) $\left\{z_{k}=a_{k}+b_{k}i|k\in \mathrm{win}\right\}$, max pooling independently selects the maximum value for both the real and imaginary parts, whereas average pooling calculates the arithmetic mean. Max pooling and average pooling can be defined as

(26) $$\begin{array}{cc} \mathrm{MaxPool}(z_{k}) & =\max_{k\in \mathrm{win}}z_{k}=\max\left(a_{k}\right)+i\;\cdot \;\max\left(b_{k}\right) \end{array}$$

(27) $$\begin{array}{cc} \mathrm{AveragePool}(z_{k}) & =\frac{1}{\left|\mathrm{win}\right|}\sum_{k\in \mathrm{win}}z_{k}=\frac{1}{\left|\mathrm{win}\right|}\sum_{k\in \mathrm{win}}a_{k}+i\;\cdot \;\frac{1}{\left|\mathrm{win}\right|}\sum_{k\in \mathrm{win}}b_{k} \end{array}$$

In the domain of signal processing, the max pooling operation, as a feature dimension reduction method, derives its physical significance primarily from its ability to enhance locally salient features. This approach effectively accentuates locally prominent features exhibiting strong responses, a characteristic of considerable value in UAV RF signal recognition applications. Specifically, within the domain of UAV RF signal processing, this method preserves both the amplitude and phase information of the RF signal from the target.

The primary benefit of average pooling is attributable to its capacity for spatial smoothing, a property that is particularly advantageous for the processing of complex signals. The process of averaging serves to effectively suppress random noise in local regions while preserving the overall energy distribution characteristics of the signal. From an implementation perspective, complex average pooling is equivalent to performing standard real-valued average pooling operations separately on the real and imaginary components of the feature map. This approach not only is computationally efficient but, more importantly, maintains the continuity of phase information in the output. In particular, the phase value of the output feature is defined as the weighted average of all complex sample phases within the specified window. This property makes it particularly suitable for applications that require strict phase consistency, such as coherent signal processing and RF data analysis of UAVs.

### 3.3. Classifier

The classifier module is composed of an FC layer and an output layer, a design that is imperative for processing complex features. The input to the proposed CV-CNN model is a flattened complex feature vector $z\in R^{2N}$, composed of the real part $a\in R^{N}$ and the imaginary part $b\in R^{N}$. For instance, if the real and imaginary parts each possess 128 dimensions, the input *z* will have a dimension count of 256. The parameter configuration for this layer is defined by the weight matrix $W\in R^{M\;\times \;2N}$, which is a matrix of real numbers with dimensions M × 2N, and the bias vector $b\in R^{M}$, which is a real number with dimensions *M*. The output dimension is denoted by *M*.

In the actual implementation process, the real part *a* and the imaginary part *b* undergo independent matrix multiplication operations (i.e., Wa and Wb), with the final results being merged. This design approach of separating computations effectively preserves the physical significance of complex features, including both magnitude and phase information. It is particularly well-suited for processing frequency-domain signals or the output features of complex convolutional layers.

The principal function of the FC layer is to map high-dimensional complex features (such as the output of complex convolutional layers) onto the target dimensional space (such as the number of categories in a classification task), and the separation of the real and imaginary components for computation ensures that the physical meaning of the complex features is preserved, thereby providing a suitable feature representation for subsequent classification tasks.

The output layer generates raw logits, which are converted into a probability distribution over the 25 UAV classes via the standard Softmax function. For numerical stability, the implementation employs the log-Softmax variant during training, consistent with common practice in deep learning frameworks.

### 3.4. Loss Function and Backpropagation

We adopt the standard cross-entropy loss to measure the discrepancy between predicted probabilities and ground-truth labels.

The model is trained using the Adam optimizer with a cross-entropy loss function, following standard practice for multi-class classification tasks. The initial learning rate is set to 0.001, with $\beta_{1}=0.9$ and $\beta_{2}=0.999$, as commonly used in the literature.

This optimization scheme effectively mitigates gradient oscillation by incorporating first-order and second-order moment estimation; subsequently addresses significant moment estimation bias during the initial phase through a deviation correction mechanism; and finally achieves robust convergence performance across varying parameter dimensions by employing an adaptive learning rate.

Gradient computation for complex-valued parameters is performed using Wirtinger calculus, which is natively supported in modern deep learning frameworks such as PyTorch (version 2.5.1).

This decoupled computational approach ensures that complex parameters retain their physical significance throughout the optimization process, while effectively capturing the coupling relationship between the real and imaginary parts.

## 4. Experiments and Results

This section conducts ablation experiments on the proposed CV-CNN model under ideal conditions (noise-free) using a spectrogram dataset generated from the raw IQ data within the DroneRFa dataset [29]. The impact of various parameters on the model’s performance in UAV RF signal classification was evaluated using accuracy. In addition, in order to provide a more comprehensive comparison of the performance of CV-CNN with that of traditional RV-CNN models, ablation studies were also conducted on the RV-CNN models. It is noteworthy that both RV-CNN and CV-CNN employed model structures and training logic that were identical throughout the course of the aforementioned ablation experiments.

In consideration of the pervasive presence of noise interference in practical application scenarios, subsequent research in Section 4.2 will undertake a more profound analysis of the performance differences between the two models under varying SNR conditions. This will facilitate a more comprehensive evaluation of the applicability of CV-CNN and RV-CNN for real-world applications.

### 4.1. Experimental Setup

**Figure 3:**
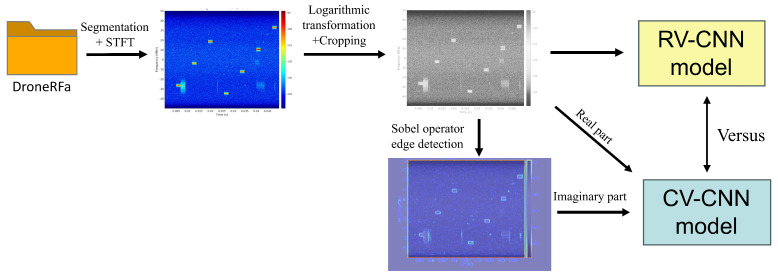
Ablation experiment flowchart.

The present study has devised a comprehensive experimental framework for CV-CNN with the objective of identifying RF signals from UAVs, as shown in Figure 3. During the construction and preprocessing of the dataset, the DroneRFA dataset was used, which comprises IQ data from 25 categories of RF signals from UAVs. The raw IQ data underwent fixed-length segmentation, with each segment containing $5\;\times \;10^{6}$ samples corresponding to a 50 ms duration at a 100 MHz sampling rate. The signal data within any two segments did not overlap. Subsequently, each segmented IQ data point underwent STFT, with the following parameters being utilized: A 1024-point Hanning window, with a 50% overlap, is utilized in conjunction with a 1024-point FFT operation, ensuring a consistent 100 MHz sampling rate. This process yielded a dataset comprising 38,480 spectrograms with a resolution of 1250 × 938.

The preprocessing workflow encompasses four critical stages: During spectrogram processing, the raw image (938 × 1250) undergoes effective region cropping; for spectrogram visualization, a 60 dB dynamic range is set (referenced to maximum power), employing a Jet color palette, with the frequency axis display range set to −50 MHz to 50 MHz to achieve centered display. Subsequently, a logarithmic transformation enhances the discernibility of low-intensity signal features. The final output comprises a 384 × 288-pixel PNG image, which serves as input for the RV-CNN and the real-part channel input for the CV-CNN. Finally, the Sobel edge detection algorithm is applied to the PNG image to generate the imaginary-part channel input, thereby constructing the complete complex representation for the CV-CNN. A comparative analysis is conducted to assess the performance of the two models under both noise-free and noisy conditions.

In terms of network architecture design, the proposed CV-CNN model comprises three core components: the complex convolutional layer strictly adheres to complex multiplication rules to ensure mathematical completeness of complex operations; the CBN layer performs independent normalization on the real and imaginary parts to maintain feature numerical stability; and the CReLU activation function implements nonlinear transformation based on complex magnitude while preserving phase information. Specifically, the network employs a dynamic feature extractor design that allows flexible adjustment of network depth based on the parameters of the ablation experiment.

The design of the ablation experiment employs a full-combination scheme of three factors in order to systematically evaluate the impact of different network configurations on performance. The first factor examines the influence of the number of convolutional layers (3, 4, 5, or 6 layers) on feature extraction capability; the second factor compares the local feature capture ability of different convolutional kernel sizes (3 × 3 and 5 × 5). The third factor contrasted two dropout regularization strategies. The “Standard” mode employed a progressively increasing dropout rate (0.3–0.8) across layers, whilst the “Reduced” mode applied a dropout rate increment strategy (0.2–0.4) exclusively to the first three layers. This multi-factor experimental design provides exhaustive empirical evidence for the optimization of model performance.

For the 25 categories of UAV RF signals, the experiment randomly partitioned the data into training, validation, and test sets at a ratio of 0.7:0.15:0.15. The implementation of the neural network model was accomplished through the utilization of the PyTorch framework (version 2.5.1, Meta Platforms, Inc., Menlo Park, CA, USA) on a Ubuntu 22.04.5 platform (Canonical Ltd., London, United Kingdom). The training, validation, and testing phases of the process were conducted on an NVIDIA RTX 3090 (NVIDIA Corporation, Santa Clara, CA, USA), utilizing the Adam optimizer and the cross-entropy loss function. The initial learning rate, weight decay, batch size, and training epoch were separately set to 0.001, $1\;\times \;10^{-5}$, 8, and 50.

### 4.2. Ablation Study on CV-CNN Architecture

In this section, the impact of network depth, kernel size, and dropout mode on the classification accuracy of the CV-CNN model was evaluated based on the results of the ablation experiments shown in Table 3.

#### 4.2.1. Impact of Network Depth

**Figure 4:**
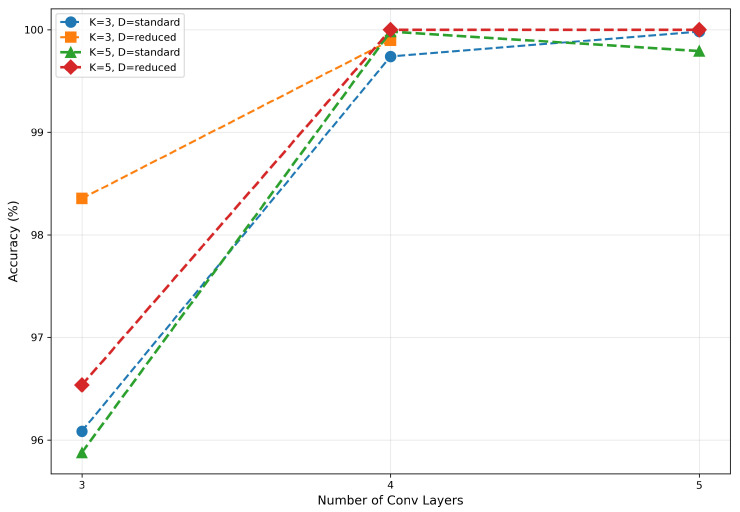
Impact of network depth on accuracy.

The experimental results indicated that network depth is the most substantial factor influencing the performance of the CV-CNN model. As shown in Figure 4, the performance of the model notably improved with an increase in network depth from 3 to 4 layers. However, when the depth was increased to five layers, the marginal benefit diminished, with performance even showing slight deterioration. In the (5, 3, Reduced) configuration, the accuracy of the model decreased to 8.52%, which demonstrates the model’s extreme sensitivity to parameter combinations within this range and its susceptibility to training instability.

**Figure 5:**
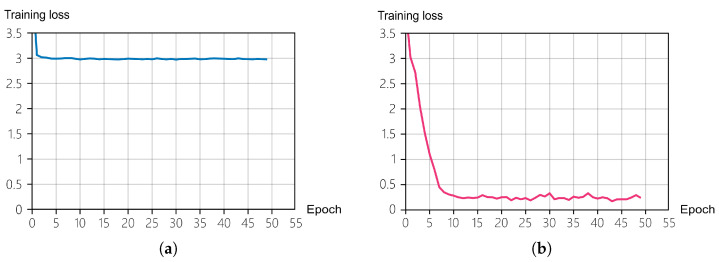
Training loss curves for (**a**) the unstable (5L, 3K, Reduced) configuration and (**b**) the stable (4L, 3K, Reduced) configuration.

This instability is primarily attributed to the combination of excessive network depth and insufficient regularization. Specifically, for deeper networks (5 or 6 layers), the “Reduced” dropout mode completely removes dropout from the final convolutional layers, which can lead to severe overfitting and gradient instability during training. This is further evidenced by the training loss curves for these configurations, which often plateau at a high value without converging, as opposed to stable models like (4L, 3K, Reduced) that show continuous improvement. A comparison of training behavior between two configurations is illustrated in Figure 5. The loss curve for the (5L, 3K, Reduced) model plateaus early, indicating a failure to learn, whereas the (4L, 3K, Reduced) model exhibits a steady decline in loss, confirming its stability.

As the depth was increased to six layers, the model demonstrated a significant deterioration in performance, with accuracy falling below 10% in the majority of configurations. This finding suggests that the network has become excessively deep, which has resulted in substantial gradient-related issues or the overfitting of the model. Consequently, subsequent analyses in this section exclude models with a depth of 6 and the (5, 3, Reduced) configuration.

#### 4.2.2. Impact of Kernel Size

**Figure 6:**
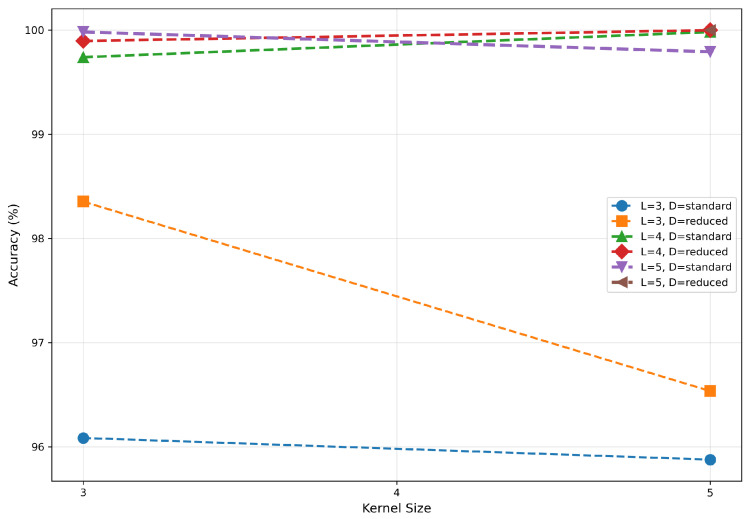
Impact of kernel size on accuracy.

The impact of kernel size on CV-CNN performance is relatively minor, exhibiting no discernible pattern, as illustrated in Figure 6. When the network depth was set at 3, models utilizing 3 × 3 convolutional kernels exhibited marginally superior performance in comparison to those employing 5 × 5 kernels. At a depth of 4, 5 × 5 kernel-based models demonstrated slightly superior performance compared to their 3 × 3 counterparts. However, at a depth of 5, the 3 × 3 kernel-based models once again exhibited slight superiority over the 5 × 5 kernel-based models, suggesting a transition in the relative effectiveness of the two kernels as the network depth increases. This phenomenon suggests that the influence of kernel size may be modulated by interactions with other parameters, rather than being an independent dominant factor.

#### 4.2.3. Impact of Dropout Mode

**Figure 7:**
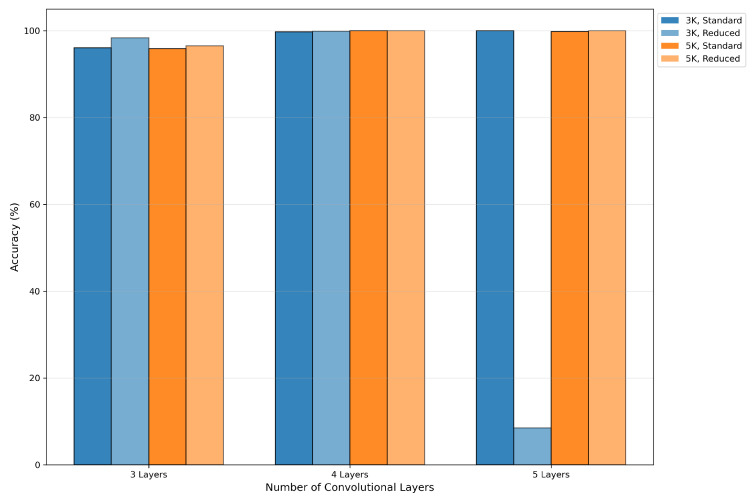
Impact of dropout mode on accuracy.

The impact of dropout mode on model performance is highly correlated with network depth, as shown in Figure 7. When model complexity was moderate (network depth = 3, 4), the ’Reduced’ mode often yielded a slight performance improvement, such as an increase from 96.08% for (3, 3, Standard) to 98.35% for (3, 3, Reduced). However, as the complexity of the model increased, the ’Reduced’ mode was shown to significantly exacerbate overfitting due to insufficient regularization strength, leading to a performance collapse. For instance, the configuration (5, 3, Reduced) attained an accuracy of merely 8.52%.

### 4.3. Performance Comparison Under Noise-Free Conditions

This section drew parallels between the performance of CV-CNN and RV-CNN models operating under identical experimental and training protocols in the absence of noise, as illustrated in Table 4.

**Figure 8:**
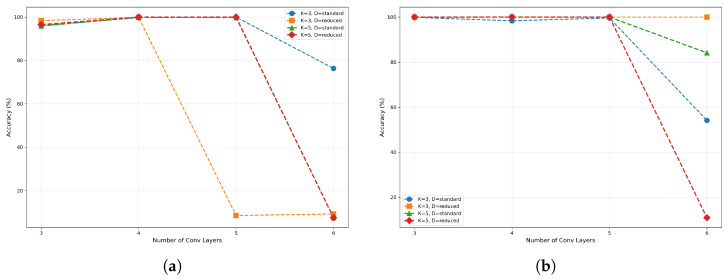
Performance comparison of (**a**) CV-CNN and (**b**) RV-CNN at different network depths.

Comparison of the performance of the two model types reveals that both can achieve near-100% accuracy under optimal configurations (e.g., network depth = 4 or 5 in most cases), indicating comparable theoretical upper limits. However, RV-CNN requires lower parameter tuning costs to achieve equivalent performance and demonstrates significant advantages in terms of model stability. As shown in Figure 8, CV-CNN demonstrated variability in performance when the network depth ranged from 3 to 5, while RV-CNN exhibited consistent and superior performance. For instance, under the configuration (5L, 3K, Reduced), RV-CNN achieved 100% accuracy, whereas CV-CNN registered only 8.52%. Furthermore, RV-CNN demonstrated greater fault tolerance to increased network depth, as shown in Figure 8b. When the network depth was designated as 6, the performance of CV-CNN was significantly compromised (often falling below 10% accuracy), while RV-CNN exhibited notable resilience with certain configurations. For instance, under (6L, 3K, Standard) and (6L, 5K, Standard) configurations, RV-CNN achieved accuracies of 99.98% and 84.13%, respectively.

**Table 4:** Comparison of accuracy between CV-CNN and RV-CNN models.

Network Depth	Kernel Size	Dropout Mode	CV-CNN Accuracy (%)	RV-CNN Accuracy (%)
3	3	Standard	96.08	99.88
3	3	Reduced	98.35	99.98
3	5	Standard	95.88	99.97
3	5	Reduced	96.53	100
4	3	Standard	99.74	98.35
4	3	Reduced	99.9	100
4	5	Standard	99.98	100
4	5	Reduced	100	100
5	3	Standard	99.98	99.62
5	3	Reduced	8.52	100
5	5	Standard	99.79	100
5	5	Reduced	100	100
6	3	Standard	76.33	99.98
6	3	Reduced	9.23	54.12
6	5	Standard	7.52	84.13
6	5	Reduced	7.52	10.93

In conclusion, the RV-CNN models offer enhanced stability and resistance to overfitting, while exhibiting comparable peak performance to the CV-CNN models. This renders the RV-CNN models a more reliable choice.

However, it should be noted that the aforementioned conclusions are obtained under ideal experimental conditions, devoid of noise. In view of the pervasive presence of noise interference in practical application scenarios, we will further analyze the performance differences between the two models under varying SNRs in the subsequent section. This extensive investigation will provide a more comprehensive assessment of the suitability of CV-CNN and RV-CNN for real-world applications.

### 4.4. Robustness Evaluation Under Varying SNR Conditions

To rigorously evaluate the robustness of the proposed CV-CNN under realistic electromagnetic interference, we conduct a series of controlled experiments across a wide SNR range from −20 dB to 20 dB. These experiments are designed to answer three key questions: (1) How does CV-CNN compare against conventional real-valued CNNs that only use magnitude spectrograms? (2) Is the observed low-SNR advantage attributable to the complex-domain arithmetic itself, rather than the input representation? (3) How does CV-CNN perform relative to representative deep learning and traditional machine learning baselines? The following subsections address these questions through progressively refined comparisons.

#### 4.4.1. Performance Comparison Between CV-CNN and RV-CNN Under Varying SNR

A systematic comparison was made between the recognition accuracy of the CV-CNN model and the traditional RV-CNN model under varying SNRs through experimental evaluation. The performance of both models was comprehensively assessed across an SNR range from −20 dB to 20 dB. As indicated by the findings in Section 4.3, both the CV-CNN and RV-CNN models demonstrated substantial degradation when the network depth reached six layers. Consequently, we abandoned analysis of models with a depth of six layers.

**Figure 9:**
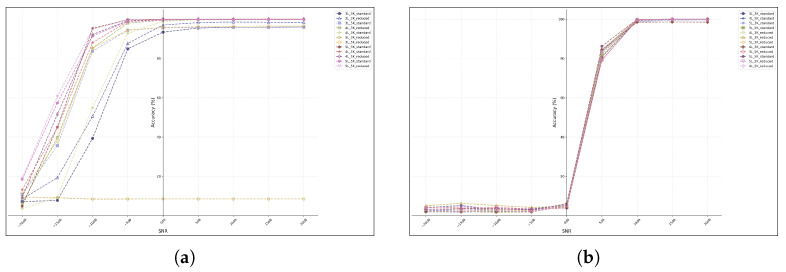
Accuracy comparison of (**a**) CV-CNN and (**b**) RV-CNN at different SNRs.

The experimental results revealed a conspicuous discrepancy in performance between the two models, particularly in regions characterized by low SNR, as shown in Figure 9. At extremely low SNR levels (−20 dB to −10 dB), the two models demonstrated fundamental discrepancies in their performance. As shown in Figure 9a, all configurations of the CV-CNN model demonstrated substandard performance at −20 dB. However, certain models, including the (5L, 5K, Standard) and (5L, 5K, Reduced) models, exhibited foundational classification capabilities, attaining accuracy rates that significantly exceeded random guessing levels. When SNR improved to −10 dB, the CV-CNN models demonstrated substantial performance gains, with most configurations achieving accuracy rates exceeding 80%. When subject to low-SNR conditions that were identical in all respects, failure of the RV-CNN models was complete, as shown in Figure 9b. At both −20 dB and −10 dB, the RV-CNN models demonstrated an accuracy rate of less than 10%, indicating an inability to extract effective features for classification in the presence of strong noise.

Within the SNR range of −10 dB to 0 dB, the CV-CNN demonstrated its most pronounced performance advantage. At an SNR of 0 dB, the CV-CNN approached near-complete convergence. The majority of model configurations achieved accuracy rates in excess of 95%, with configurations such as (5L, 5K, Standard) and (4L, 5K, Standard) attaining or approaching 100% accuracy. In contrast, the performance of RV-CNN in this range exhibited a considerable delay. Even at 0 dB, the accuracy of all RV-CNN models remained below 10%, indicating a substantial discrepancy compared to CV-CNN models.

As SNR continued to increase, the performance of the RV-CNN models began to improve rapidly. Beyond an SNR exceeding 10 dB, the accuracy of all CV-CNN and RV-CNN models approached saturation, reaching or approaching the ultimate performance threshold of 100%. This finding indicated that, in conditions of high SNR, both models demonstrate the capacity to leverage the unique signal characteristics in their entirety.

#### 4.4.2. Controlled Comparison Between CV-CNN and Dual-Channel RV-CNN with Identical Inputs

Section 4.4.1 demonstrated that the CV-CNN outperforms the standard magnitude-only RV-CNN. However, both models utilize different input representations: the RV-CNN processes only the logarithmic power spectral density, whereas the CV-CNN incorporates an additional Sobel edge map as the imaginary component. Consequently, it remains unclear whether the observed performance gain is attributable to the complex-valued processing itself or simply to the richer input representation. To isolate the effect of complex arithmetic, we design a controlled experiment where both models receive identical dual-channel inputs, the logarithmic PSD and Sobel edge map, and share the same architectural hyperparameters. This setup allows us to directly attribute any performance difference to the underlying arithmetic domain (complex vs. real).

We implemented a dual-RV-CNN that uses the exact same input representation as our CV-CNN: logarithmic PSD as channel 1 and Sobel edge map as channel 2. Both models share identical architecture hyperparameters (4L, 5K, Reduced), and were trained and evaluated under the same protocol.

**Figure 10:**
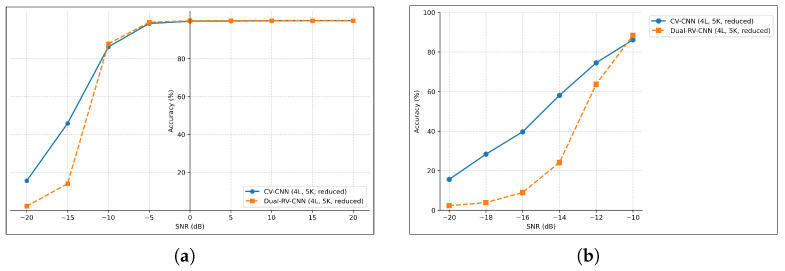
Accuracy comparison of CV-CNN and dual-RV-CNN across an SNR range from (**a**) −20 dB to 20 dB (in 5 dB steps), and (**b**) −20 dB to −10 dB (in 2 dB steps).

To provide a complete picture, we also include the full-SNR-range comparison (Figure 10) for this representative configuration. This clearly illustrates that while the two models perform comparably in high-SNR regimes, the CV-CNN exhibits significantly superior robustness under severe noise conditions.

As shown in Figure 10a, both models achieve near-perfect accuracy above −10 dB SNR. However, in the critical low-SNR regime (−20 dB to −10 dB), the CV-CNN demonstrates a clear advantage. To investigate this further, we refined the evaluation granularity from 5 dB to 2 dB steps in this range. The results in Figure 10b confirm that the CV-CNN consistently outperforms the dual-RV-CNN, with 15.58% vs. 2.20% at −20 dB, and 45.86% vs. 14.03% at −15 dB, despite identical inputs. This indicates that the benefit arises not from the input representation alone, but from the intrinsic noise resilience of complex-domain arithmetic during feature fusion.

#### 4.4.3. Comparison with State-of-the-Art Deep and Traditional Machine Learning Models

To provide a more comprehensive evaluation of the proposed CV-CNN’s performance, we extended our baseline comparisons beyond RV-CNN to include several widely used deep learning architectures for image classification (ResNet18 and EfficientNet-B0) and traditional machine learning methods (SVM, Random Forest, XGBoost, KNN). We also implemented LSTM and GRU models, which are commonly applied to time-series data. All models were trained and evaluated on the same preprocessed DroneRFa dataset using identical experimental protocols. The results under noise-free conditions are summarized in Table 5.

**Figure 11:**
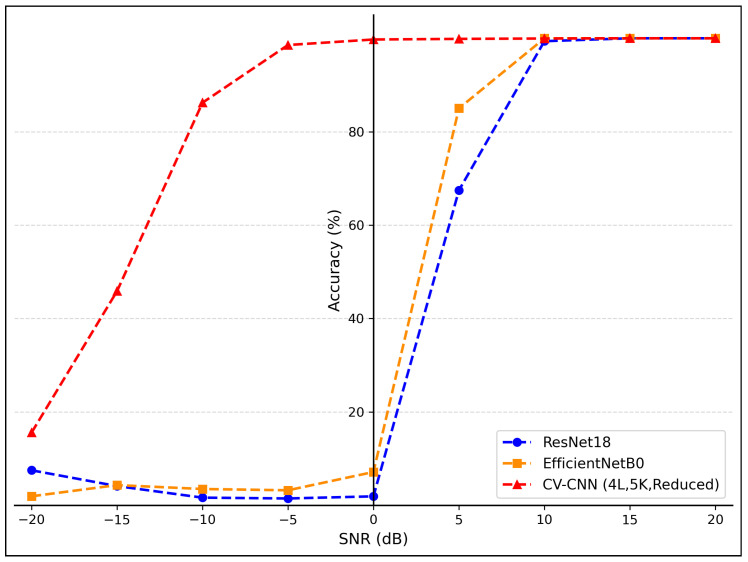
Accuracy comparison of CV-CNN (4L, 5K, Reduced), ResNet18, and EfficientNet-B0 across an SNR range from −20 dB to 20 dB.

As shown in Table 5, while ResNet18 and EfficientNet-B0 achieve perfect accuracy under ideal conditions, they exhibit significantly lower robustness to noise compared to CV-CNN, as demonstrated in Figure 11. In contrast, LSTM and GRU, despite their suitability for sequential data, perform poorly on our spectrogram inputs, likely due to the loss of temporal structure during STFT preprocessing. Traditional machine learning methods also show subpar performance, primarily because they rely on handcrafted features that fail to capture the complex, high-dimensional patterns present in RF spectrograms. This highlights the advantage of end-to-end deep learning models, particularly those designed to process the full complex-domain structure of the signal.

In summary, under the experimental conditions and dataset employed in this study, the CV-CNN demonstrated notably superior performance over RV-CNN under low-SNR conditions. CV-CNN has been shown to maintain effective feature extraction and classification capabilities even in extremely low-SNR environments, where RV-CNN has been demonstrated to fail, thus suggesting potential robust noise resilience of CV-CNN. Concurrently, CV-CNN exhibited a higher performance threshold, achieving near-perfect classification performance at 0 dB SNR, whereas RV-CNN remained virtually inoperable at this SNR level. While these results highlight the promise of complex-domain modeling for RF signal recognition under noise, it should be acknowledged that they are based on a single dataset and preprocessing pipeline. Generalization to other scenarios warrants further investigation.

In practical applications, when the anticipated SNR in the working environment is below 10 dB, the CV-CNN architecture should be prioritized. Conversely, in high-SNR environments (more than 10 dB), the performance discrepancy between the two models is significantly reduced, enabling selection based on computational efficiency and other factors. The anomalous performance observed at five layers and the comprehensive degradation at six layers provide crucial avenues for future research. This finding necessitates further investigation into the optimal depth constraints for CV-CNN and the underlying mechanisms governing their interaction with regularization strategies.

### 4.5. Real-Time Inference Performance Evaluation

The practical deployment of UAV recognition systems in real-world scenarios demands not only high accuracy but also low-latency inference and efficient resource utilization. To assess the operational viability of the proposed CV-CNN model (configured as 4L, 5K, Reduced), we conduct a dedicated evaluation of its inference characteristics under conditions that simulate real-time monitoring. This section investigates two critical aspects: (1) whether the model meets the time-sensitive requirements of real-time UAV detection, specifically, in terms of latency and throughput for processing continuous RF data streams, and (2) its computational footprint, which informs the potential for future deployment on resource-constrained, field-deployable platforms such as embedded devices.

Specifically, we measure three key metrics under identical experimental conditions: (1) average inference time per 50 ms RF signal segment (corresponding to one spectrogram input); (2) throughput in terms of samples processed per second; and (3) GPU memory consumption during inference. All tests are conducted on an NVIDIA RTX 3090 GPU using the PyTorch framework, with input data preprocessed as described in Section 4.1.

**Figure 12:**
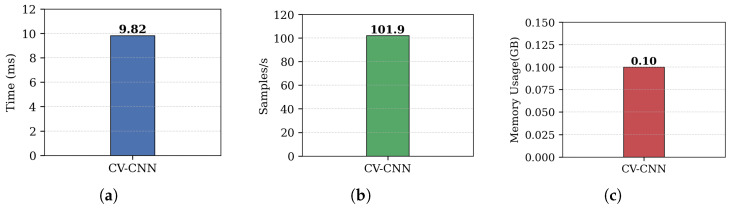
Inference performance of the (4L, 5K, Reduced) CV-CNN model. (**a**) Average inference time per sample; (**b**) throughput in samples per second; (**c**) GPU memory consumption during inference.

The results, summarized in Figure 12, indicate that the model achieves an average inference latency of 9.82 ms per sample, enabling a throughput of 101.9 samples per second. The peak GPU memory footprint during inference is 0.1 GB. These preliminary results demonstrate that the current implementation operates with low latency and moderate resource usage, suggesting its potential suitability for real-time scenarios. The ability to process over 100 samples per second on a single GPU is a strong indicator of feasibility for many operational contexts where rapid detection is required.

While this study focuses on algorithmic validation, we recognize that actual deployment in operational UAV monitoring systems requires further engineering optimizations, including model quantization, hardware-aware acceleration, and integration with continuous RF data streaming pipelines. These aspects will be thoroughly investigated in our future work to realize a fully functional real-time CV-CNN-based UAV recognition system.

Regarding the suitability for deployment on embedded devices, we acknowledge that our current evaluation is conducted on a high-performance NVIDIA RTX 3090 GPU, which represents a desktop/server-grade platform rather than a field-deployable embedded system. While the measured metrics (9.82 ms latency, 0.1 GB memory) are promising and suggest that the model’s computational footprint is not prohibitively large, direct deployment on resource-constrained embedded hardware (e.g., ARM-based SoCs or FPGAs) requires further optimization. These optimizations may include model quantization, pruning, knowledge distillation, and the use of specialized inference engines (e.g., TensorRT, OpenVINO). The low latency and moderate memory usage observed in our preliminary tests provide a strong foundation for such future work, indicating that the CV-CNN architecture is not inherently incompatible with embedded deployment. However, a definitive assessment of its feasibility on specific embedded platforms will require targeted benchmarking and hardware-aware implementation, which constitutes a critical next step beyond the scope of this algorithmic validation study.

## 5. Discussions

The experimental results presented in this study demonstrate that the proposed CV-CNN achieves significantly enhanced robustness under low-SNR conditions compared to its real-valued counterpart, despite both models receiving identical dual-channel inputs (log-PSD and Sobel edge map). This advantage aligns with theoretical expectations from the complex-valued deep learning literature [21,24], which posits that complex-domain operations impose stronger algebraic constraints and better preserve the intrinsic structure of signals originally defined in the complex plane. Our findings corroborate this hypothesis in the specific context of UAV RF signal recognition, where phase-like structural cues, encoded via edge gradients, complement magnitude information to sustain discriminability even when SNR drops below −10 dB. It is worth noting that while other recent works have explored complex-valued models for RF signal classification [31], our approach uniquely focuses on leveraging a hybrid magnitude–edge representation to achieve superior noise resilience, rather than relying on raw data.

Nevertheless, several important limitations must be acknowledged. First, the “phase” component in our complex representation is not the true RF phase but a surrogate derived from Sobel edge detection on the power spectrogram. Although this design empirically improves low-SNR performance, it discards the original hardware-specific phase signatures (e.g., oscillator drift). This loss may limit the model’s ability to discriminate between devices with subtle RF fingerprint differences. Second, the CV-CNN exhibits high sensitivity to architectural hyperparameters, particularly network depth. As shown in Table 3, configurations beyond four layers often suffer from severe training instability, leading to catastrophic accuracy drops (e.g., 8.52% for “5L, 3K, Reduced”). This suggests that existing regularization strategies (e.g., dropout) may be insufficient for deeper complex networks, warranting future investigation into complex-specific normalization or initialization schemes. Potential directions include adopting orthogonal or unitary initialization for complex weights [24], exploring complex BatchNorm variants that jointly normalize magnitude and phase [22], or integrating spectral regularization to stabilize training dynamics in deep CV-CNNs.

Third, all experiments are conducted on a single dataset (DroneRFa) with a fixed preprocessing pipeline (STFT to log-PSD and to Sobel). The generalizability of our approach to other RF datasets, modulation types, or time–frequency representations (e.g., CWT) remains unverified. Finally, the current framework is not fully end-to-end; it relies on handcrafted feature construction rather than learning the optimal complex representation directly from raw IQ data. While this hybrid approach balances interpretability and performance, an end-to-end complex model may unlock further gains if training stability challenges can be overcome.

Despite these limitations, this work provides compelling evidence that complex-valued modeling, when carefully designed, can substantially improve the noise resilience of RF-based UAV recognition systems, offering a promising direction for future research in complex-domain signal intelligence. Looking ahead, the insights from collaborative multi-agent systems [32] suggest that robust RF signal recognition could serve as a foundational component for future cooperative UAV traffic management and swarm intelligence frameworks.

## 6. Conclusions

This study aims to implement CV-CNN in recognizing RF signals from UAVs. In order to leverage phase information, a CV-CNN incorporating complex convolutional layer, CBN, CReLU, complex pooling, and complex backpropagation algorithms is proposed. A series of ablation experiments was conducted on the DroneRFa dataset, with the aim of comparing the performance of the proposed CV-CNN with that of an RV-CNN sharing the same architecture. Evaluated on the 25-class DroneRFa dataset, the proposed CV-CNN achieves 100.00% accuracy under noise-free conditions. Crucially, it demonstrates significantly superior robustness in low-SNR regimes: at −20 dB SNR, it attains 15.58% accuracy, over seven times higher than a dual-channel RV-CNN (2.20%) with identical inputs; at −15 dB, it reaches 45.86% versus 14.03%. This research provides a foundation for the broad application of CV-CNN in the domain of UAV RF signal recognition.

Importantly, the advantage of CV-CNN does not stem from preserving the original RF phase, which is lost during PSD computation. Instead, it arises from the model’s ability to process a magnitude–edge complex representation through genuine complex arithmetic, which yields superior low-SNR robustness compared to real-valued alternatives. The experimental results suggest that, within the scope of the DroneRFa dataset and the adopted preprocessing strategy, the CV-CNN exhibits enhanced robustness and interference resistance in comparison to RV-CNN of identical architecture, maintaining high recognition accuracy even under low-SNR conditions.

Furthermore, comparative experiments with state-of-the-art image classification networks (ResNet18, EfficientNet-B0) and traditional machine learning methods confirm that the proposed CV-CNN offers not only high accuracy but also exceptional robustness under challenging low-SNR conditions.

While these results are promising, it should be noted that practical deployment may be constrained by the need for GPU-supported complex arithmetic and dependence on controlled RF data acquisition conditions (e.g., fixed sampling rate and known frequency bands), which could affect adaptability in highly dynamic electromagnetic environments.

## Figures and Tables

**Table 1:** Architecture configuration of the (4L, 3K, Reduced) CV-CNN model.

Layer Type	Layer Parameter	Activation
Input	1 × 2 × 384 × 288	–
CvConv1 + CBN	k = 3 × 3, s = 1, p = 1 → 1 × 64 × 384 × 288	CReLU
MaxP	MaxPool 2 × 2 → 1 × 64 × 192 × 144	–
Dropout1	p = 0.3	–
CvConv2 + CBN	k = 3 × 3, s = 1, p = 1 → 1 × 128 × 192 × 144	CReLU
MaxP	MaxPool 2 × 2 → 1 × 128 × 96 × 72	–
Dropout2	p = 0.4	–
CvConv3 + CBN	k = 3 × 3, s = 1, p = 1 → 1 × 256 × 96 × 72	CReLU
MaxP	MaxPool 2 × 2 → 1 × 256 × 48 × 36	–
Dropout3	p = 0.5	–
CvConv4 + CBN	k = 3 × 3, s = 1, p = 1 → 1 × 256 × 48 × 36	CReLU
AvgP	AdaptiveAvgPool 6 × 6 → 1 × 256 × 6 × 6	–
Flatten	1 × 9216	–
FC1	9216 → 1024	ReLU
DropoutFC	p = 0.5	–
FC2	1024 → 512	ReLU
DropoutFC	p = 0.5	–
FC3/Output	512 → 25	Softmax

**Table 2:** Distinction between complex-valued and real-valued convolution.

Aspect	Complex-Valued Convolution	Real-Valued Convolution
Input/output	Complex number	Real number
Number of parameters	4 times real-valued	Relatively few
Phase processing	Preserving phase	Phase loss
Frequency domain	Correlation modeling	Magnitude-only
Computational complexity	Higher	Lower

**Table 3:** Accuracy of CV-CNN model under different parameter configurations.

Network Depth	Kernel Size	Dropout Mode	Accuracy (%)
3	3	Standard	96.08
3	3	Reduced	98.35
3	5	Standard	95.88
3	5	Reduced	96.53
4	3	Standard	99.74
4	3	Reduced	99.90
4	5	Standard	99.98
4	5	Reduced	100.00
5	3	Standard	99.98
5	3	Reduced	8.52
5	5	Standard	99.79
5	5	Reduced	100.00
6	3	Standard	76.33
6	3	Reduced	9.23
6	5	Standard	7.52
6	5	Reduced	7.52

**Table 5:** Accuracy of various models under noise-free conditions on the DroneRFa dataset.

Model	Accuracy (%)
CV-CNN (4L, 5K, Reduced)	100.00
ResNet18	100.00
EfficientNet-B0	100.00
LSTM	50.33
GRU	51.20
SVM	45.48
Random Forest	69.63
XGBoost	73.20
KNN	49.74

## Data Availability

The original contributions presented in this study are included in the article. Further inquiries can be directed to the corresponding author.

## Abbreviations

The following abbreviations are used in this manuscript:

UAV	Unmanned aerial vehicle
RF	Radio frequency
PSD	Power spectral density
CNN	Convolution neural network
CV-CNN	Complex-valued convolution neural network
RV-CNN	Real-valued convolution neural network
SNR	Signal-to-noise ratio
CBN	Complex batch normalization
CReLU	Complex rectified linear unit
STFT	Short-Time Fourier Transform
FC	Fully connected layer
SVM	Support Vector Machine
KNN	K Nearest Neighbors
LSTM	Long Short-Term Memory
GRU	Gated Recurrent Unit
